# Unique characteristics of the J-domain proximal regions of Hsp70 cochaperone Apj1 in prion propagation/elimination and its overlap with Sis1 function

**DOI:** 10.3389/fmolb.2024.1392608

**Published:** 2024-04-24

**Authors:** Samantha J. Ganser, Bridget A. McNish, Gillian L. Schwanitz, John L. Delaney, Bridget A. Corpus, Brenda A. Schilke, Anup K. Biswal, Chandan Sahi, Elizabeth A. Craig, Justin K. Hines

**Affiliations:** ^1^ Department of Chemistry, Lafayette College, Easton, PA, United States; ^2^ Department of Biochemistry, University of Wisconsin–Madison, Madison, WI, United States; ^3^ Department of Biological Sciences, Indian Institute of Science Education and Research Bhopal, Bhopal, India

**Keywords:** chaperone, amyloid, Hsp104, Hsp40, J-protein

## Abstract

J-domain proteins (JDPs) are obligate cochaperones of Hsp70s. The Class A JDP Apj1 of the yeast cytosol has an unusually complex region between the N-terminal J-domain and the substrate binding region—often called the G_rich_ or GF region in Class A and B JDPs because of its typical abundance of glycine. The N-terminal 161-residue Apj1 fragment is known to be sufficient for Apj1 function in prion curing, driven by the overexpression of Hsp104. Further analyzing the N-terminal segment of Apj1, we found that a 90-residue fragment that includes the 70-residue J-domain and the adjacent 12-residue glutamine/alanine (Q/A) segment is sufficient for curing. Furthermore, the 121-residue fragment that includes the G_rich_ region was sufficient to not only sustain the growth of cells lacking the essential Class B JDP Sis1 but also enabled the maintenance of several prions normally dependent on Sis1 for propagation. A J-domain from another cytosolic JDP could substitute for the Sis1-related functions but not for Apj1 in prion curing. Together, these results separate the functions of JDPs in prion biology and underscore the diverse functionality of multi-domain cytosolic JDPs in yeast.

## 1 Introduction

Hsp70s are ubiquitous and promiscuous molecular chaperone proteins that accomplish myriad functions around the cell by binding and releasing client polypeptides in an ATP-dependent cycle (Rosenzweig et al., 2019). The ability of Hsp70s to locate and act upon various cellular targets arises from their pairing with an obligate cochaperone, a J-domain protein (JDP), also referred to as a J-protein or Hsp40 (Kampinga et al., 2019). All JDPs have a canonical ∼70-residue domain called a J-domain, which, upon binding Hsp70, can stimulate its ATPase activity (Kampinga and Craig, 2010). ATP turnover then enhances Hsp70 client-peptide binding. Many JDPs can also bind client proteins and direct them to Hsp70s (Kampinga and Craig, 2010).

**FIGURE 1:**
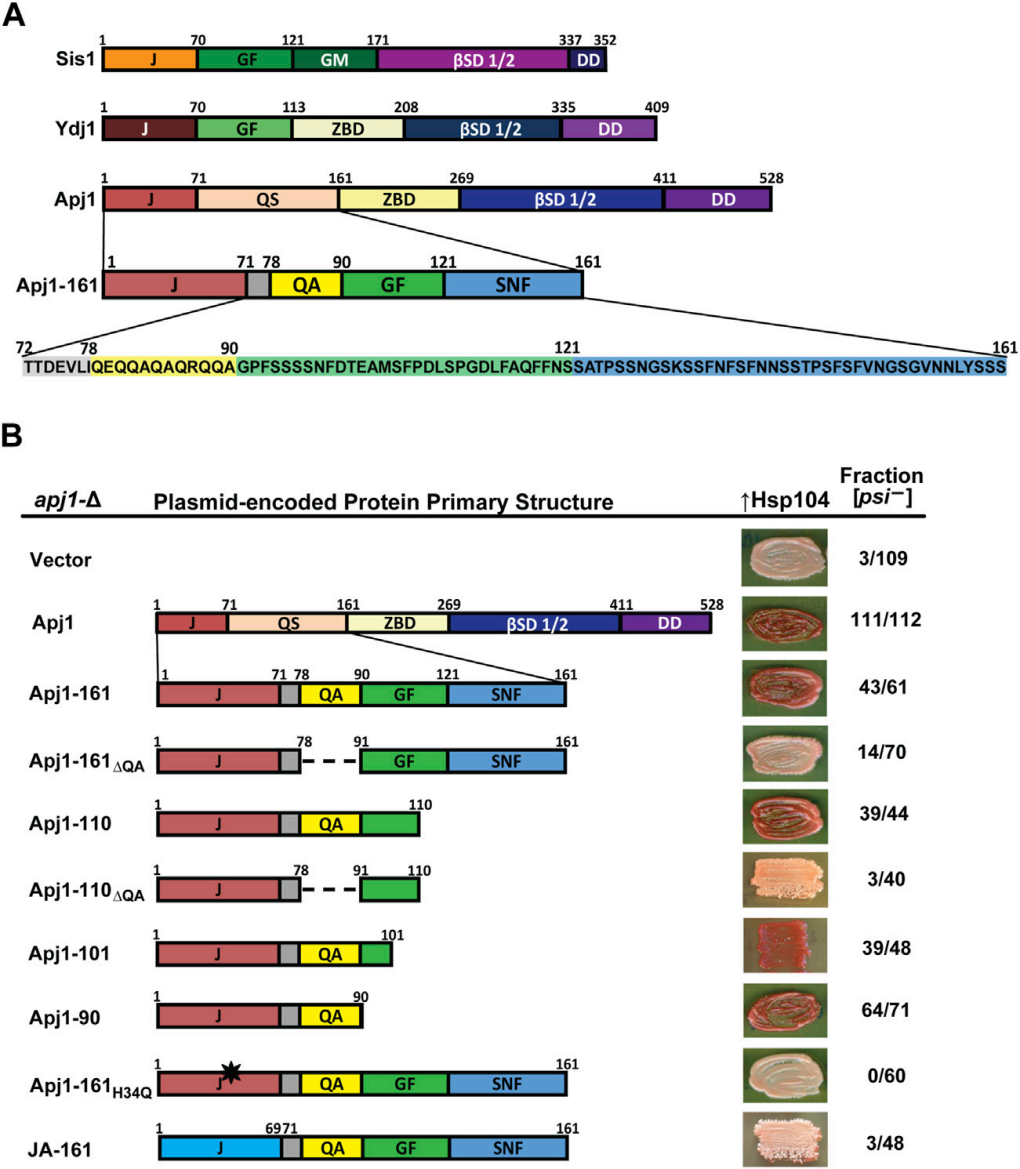
A functioning J-domain and the QA repeat region are sufficient to support Hsp104-mediated [*PSI* ^+^] curing. **(A)** Comparison of primary structures of Sis1, Ydj1, and Apj1. Upon examination of the sequence, the QS region was divided into subregions and color coded for clarity. Protein regions are denoted as follows: J, J-domain; GF, glycine/phenylalanine-rich region; GM, glycine/methionine-rich region; βSD 1/2, β-sandwich domains 1 and 2; DD, dimerization domain; ZBD, zinc-binding domain; QS, glutamine/serine-rich region. Subregions of the Apj1 QS region are denoted as follows: QA, glutamine/alanine-rich region; GF, glycine/phenylalanine-rich region; SNF, serine/asparagine/phenylalanine-rich region. **(B)** Apj1 domain requirements for Hsp104-dependent elimination of the prion [*PSI* ^+^]^Sc4^. [*PSI* ^+^]^Sc4^ cells lacking genomic *APJ1* were transformed with plasmids expressing Apj1 constructs. Cells were then transformed again with a plasmid overexpressing Hsp104 (*GPD-Hsp104*). Primary sequence diagrams of Apj1 constructs used in the experiments are illustrated on the left. Dashed lines indicate where a region has been deleted. The black star indicates a point mutation. Color phenotype assays are shown for representative transformants (right). The number of transformants becoming [*psi* ^−^] (cured) is given as a fraction of the total examined (*n* ≥ 40).

JDPs are grouped into three classes—A, B, and C—depending on their domain structure (Marszalek et al., 2024). Class A and B JDPs share a similar architecture, while JDPs possessing any other architecture are placed in Class C. Class A and B JDPs have an N-terminal, 4-helix J-domain typically followed by a glycine-rich region and two β-sandwich domains, followed by a C-terminal dimerization domain. By definition, members of Class A have a zinc-binding domain protruding from the N-terminal β-sandwich, while members of Class B do not (see Figure 1A) (Marszalek et al., 2024). Four Class A/B JDPs are present in the cytosol of *Saccharomyces cerevisiae* (Sahi and Craig, 2007); three belong to Class A: Ydj1, Apj1, and Xdj1. Apj1 and Xdj1 arose during the fungal lineage via two separate duplications of the *YDJ1* gene (Sahi et al., 2013). Ydj1, the most abundant of the four, is not essential, but *ydj1*-*∆* cells grow poorly (Caplan and Douglas, 1991). *apj1*-*∆* and *xdj1*-*∆* cells have much more subtle defects under normal growth conditions (Sahi and Craig, 2007). Although Apj1 is a descendent of Ydj1, it has an atypical region adjacent to the J-domain—more complex in sequence composition than is typical for Class A/B JDPs. There is only one Class B JDP in the cytosol, Sis1, which is essential and cannot be replaced by the overexpression of any other full-length yeast JDP (Sahi and Craig, 2007). This study focuses on two JDPs that have been linked to yeast prions: Apj1 and Sis1.

Prions are self-propagating aggregates of misfolded protein, most commonly in the form of amyloids (Wickner et al., 2011). At least 10 amyloid-forming prions are present in *S. cerevisiae*, the best studied being the prion [*PSI* ^+^] (Liebman and Chernoff, 2012). Prions exhibit amyloid polymorphisms, called “strains” in mammals and “variants” in yeast, in which the same prion-forming protein can form distinct, stable amyloid structures (Killian et al., 2019). Variants of [*PSI* ^+^] are generally denoted as “strong” and “weak”, describing both the strength of the corresponding phenotype and the stability of the prion in cell populations (Liebman and Chernoff, 2012).

Yeast prions are propagated within cell populations by a fragmentation mechanism that minimally requires the chaperone proteins Hsp104, Hsp70, and the Class B JDP Sis1 (Higurashi et al., 2008; Killian and Hines, 2018). Hsp104, a protein disaggregase of the AAA+ class that was originally characterized by Susan Lindquist and colleagues (Parsell et al., 1991; Parsell et al., 1994; Wendler et al., 2007), fragments large prion aggregates with the assistance of Sis1 and Hsp70 (Liebman and Chernoff, 2012). Prion fragmentation results in smaller and more numerous “seeds” that can be inherited by daughter cells during cell division (Cox et al., 2003). When Hsp104 is ectopically overexpressed, [*PSI* ^+^] is quickly eliminated from cell populations in a Sis1-dependent manner (Chernoff et al., 1993; Kirkland et al., 2011; Sporn and Hines, 2015). In addition to Sis1, Apj1 (“anti-prion DnaJ”), which was first identified in a screen for proteins that cured a synthetic prion upon overexpression (Kryndushkin et al., 2003), is also required for Hsp104 curing of strong variants of [*PSI* ^+^] (Astor et al., 2018). The first 161 residues of Apj1 are sufficient to support Hsp104 curing (Berger et al., 2020). Furthermore, overexpression of either Sis1 or Apj1 can complement the absence of the other in supporting Hsp104 curing (Astor et al., 2018).

Results to date suggested that Apj1 and Sis1 may have overlapping functions in Hsp104 prion curing and that the unusually long region between the J-domain and the start of the first β-sandwich is critical in this process. This region, ending in residue 161, is rich in glycines, glutamines, and serines and has been called the QS region (Sahi and Craig, 2007) to distinguish it from the less complex G_rich_ regions generally found in Class A/B JDPs. Therefore, we further examined the N-terminal 161 residues of Apj1. Our results revealed novel functionality of two sequence elements, one in prion elimination and the other in promoting cell viability and prion propagation, demonstrating both surprising functional conservation and divergence among yeast JDPs.

## 2 Materials and methods

### 2.1 Yeast strains

Haploid *S. cerevisiae* strains derived from the W303 genetic background were used throughout this investigation. Unless otherwise noted, strains had the genotype: *trp1-1 ura3-1 leu2-3,112* *his3-11,15 ade1-14*. A strain lacking *APJ1* (*apj1:HIS3*) and bearing the well-characterized strong [*PSI* ^+^] variant [*PSI* ^+^]^Sc4^ was used for assays to test Hsp104-mediated prion elimination, as previously described (Astor et al., 2018; Berger et al., 2020). Strains harboring the prions [*PSI* ^+^]^Sc4^, [*PSI* ^+^]^Sc37^, or [*RNQ* ^+^]^STR^ and covering a *SIS1* deletion (*sis1:LEU2*) with a plasmid expressing Sis1 ([*SIS1*-*SIS1*, *URA3*]) were used for plasmid-shuffling experiments to test for prion propagation as previously described (Harris et al., 2014). To analyze yeast cell growth, cells were counted using a hemocytometer, and ten-fold serial dilutions were spotted in 5 μL drops onto selective synthetic medium (Sherman et al., 1986) and grown for 2–5 days at the indicated temperatures.

### 2.2 Plasmids

All plasmids used in this study are listed in Supplemenatry Table S1 and derived from the pRS series (Mumberg et al., 1995). Plasmids were harvested from isolated DH5α *Escherichia coli* cells using QIAprep Spin Miniprep Kit (QIAGEN, Valencia, CA). Apj1 gene constructs with and without the 3XFLAG tag sequence were made using standard molecular cloning protocols and sequenced before use.

### 2.3 Plasmid shuffling

For experiments in which Sis1 is being replaced, cells were transformed by JDP-expressing plasmids and transformants were grown on selective medium. The *URA3-*marked *SIS1* plasmid was shuffled out by growth on medium containing 5-fluoroorotic acid (5-FOA), which counter-selects against the *URA3-*marked plasmid (Boeke, et al., 1987). Loss of the *URA3*-marked plasmid was confirmed by the lack of growth on synthetic medium lacking uracil. Shuffled cells were then grown on selective medium for use in assays.

### 2.4 Gel electrophoresis and immunoblotting

For sodium dodecyl sulfate polyacrylamide gel electrophoresis (SDS-PAGE), cell pellets of 1 O.D. were collected, resuspended in 0.2 M NaOH, vortexed, and incubated at room temperature for 5 min. Cells were centrifuged at maximum speed on a tabletop minicentrifuge for 1 min, and the pellet was collected. Prior to electrophoresis, samples were suspended in 2x SDS-PAGE running buffer and boiled for 5 min before resolving in a 12.5% polyacrylamide gel at 120 V.

Semi-denaturing detergent agarose gel electrophoresis (SDD-AGE), a method for resolving detergent-resistant aggregates (Kryndushkin et al., 2003), was used to confirm the presence of prions. Cells were grown in liquid 5 mL cultures in rich YPD medium (Teknova) which is 1% (^w^/_v_) yeast extract, 2% (^w^/_v_) peptone, 2% (^w^/_v_) dextrose, and 2.4% (^w^/_v_) agar. Pellets of 4 O.D. were harvested, and cells were lysed using sterile glass beads suspended in buffer and vortexed at 4 °C with a Genie SI-D248 Disruptor Shaker (Scientific Industries). Lysates were then centrifuged at 4°C for 5 min at 2000 RPM on a tabletop minicentrifuge. They were mixed with SDD-AGE loading buffer for 7 min, and electrophoresis was performed using a 1.5% (^w^/_v_) Tris-glycine, 0.1% (^v^/_v_) SDS, agarose (SeaKem Gold Agarose) gel at 120 V.

For immunoblotting, proteins were transferred to a nitrocellulose membrane at 1 A for 1 h while being chilled in Tris-glycine/methanol buffer. To visualize aggregates, membranes were blocked with 5% (^w^/_v_) dried milk and probed with polyclonal antibodies for either Rnq1 or Sup35 (a gift from the Tuite lab).

### 2.5 Assays for prion elimination or propagation

For prion propagation, following plasmid-shuffling to replace *SIS1*, shuffled cells bearing prions were grown on selective medium at 30 °C for 2 days and repatched as needed to allow time for prion loss. Cells were then grown on rich medium without additional adenine until color development. The colonies exhibit a color phenotype on rich medium in which [*PSI* ^+^] cells appear pink and [*psi* ^−^] cells appear red. The red color results from a mutation in the adenine biosynthesis pathway. As a result, growth on rich medium without additional adenine can be used as an assay for [*PSI* ^+^] presence or elimination (Liebman and Chernoff, 2012). When necessary, prion status was confirmed with SDD-AGE. For [*RNQ* ^+^] propagation, shuffled cells were grown on selective medium for 2–3 days to allow time for prion loss, and SDD-AGE was used to determine the continued presence of [*RNQ* ^+^].

For prion loss by Hsp104 overexpression following the growth of colonies on selective medium post-transformation with the *TRP1*-marked plasmids, all strains were transformed a second time with pRS416-*GPD HSP104*, a *URA3-*marked Hsp104 overexpression vector. After colonies grew on selective medium, they were streaked to patches on selective medium, grown at 30 °C for 2 days, and repatched to rich medium without additional adenine added. Curing experiments were conducted in batches of ten transformants. Thus *n* = 40 indicates that the entire curing experiment was repeated at least four times.

## 3 Results

### 3.1 Dissection of the atypical QS region adjacent to Apj1’s J-domain

To further investigate the function of the QS region of Apj1, we examined its sequence distribution more thoroughly to inform construction of Apj1-161 variants to test their effects on *in vivo* function. After further inspection, we subdivided this region based on unique features, amino acid composition, and homology with Sis1 and Ydj1 (Figure 1A). Most striking, due to its absence in any other yeast G_rich_ region, is the presence of a 12-residue region with an imperfect QA repeat. Although Apj1 does not have a stereotypical G_rich_ region, the segment following the QA sequence is a region with a limited degree of sequence similarity with the C-terminal portion of the G_rich_ region of Ydj1 and the related region called GF in Sis1, with some key features conserved. Apj1 has the sequence DLFAQFF, while Ydj1 has DIFSQFF. Sis1 has DAFNIFSQFF, containing residues important for prion maintenance and the ability of Sis1-121 to rescue the lethality of *sis1*-∆ (Lopez et al., 2003). Residues in this region form a helix in Ydj1, Sis1, and its human ortholog, DNAJB1, referred to as “Helix V” in the literature as it is typically the predominant helix after the four helices of the J-domain (Ciesielski, et al., 2024). The remaining 40 residues comprise another low complexity region highly enriched in S, N, and F residues (38% S, 18% N, 13% F, and 8% G). The region has just one charged residue and two prolines and is denoted “SNF” in Figure 1A. BLASTP and I-TASSER searches confirm that this region has no significant sequence homology or predicted structural homology to any known protein apart from other Apj1 orthologs. We thus designated residues 1–71 as the J-domain, 79–90 as the QA region, 91–121 as the GF region, and 122–161 as the SNF region (Figure 1A). In the next sections, we describe the results of experiments testing the function of different Apj1-161 deletion variants.

### 3.2 Apj1’s J-domain and QA region, not its GF or SNF regions, are important for Hsp104 curing

We first tested the ability of Apj1-161 constructs to complement *apj1*-Δ in restoring efficient prion curing when Hsp104 is overexpressed. To do this, an *apj1-∆* strain that stably propagates the strong variant [*PSI* ^+^]^Sc4^ (Astor et al., 2018) was transformed with plasmids expressing Apj1 constructs or empty vector. In this strain, [*psi* ^−^] colonies appear red on rich medium due to a blocked adenine biosynthesis pathway, whereas [*PSI* ^+^]^Sc4^ colonies appear pink due to [*PSI* ^+^]-dependent nonsense suppression that partially restores adenine prototrophy. Importantly, propagation of the prion was unaffected by the expression of any Apj1 construct, consistent with our previous observations (Astor et al., 2018; Berger et al., 2020). We next transformed these strains with a multicopy plasmid overexpressing Hsp104 (*GPD-HSP104*) that normally results in rapid [*PSI* ^+^]^Sc4^ curing in otherwise wild-type strains (Astor et al., 2018). As previously observed, *apj1-∆* cells carrying only an empty vector were protected from Hsp104 curing (3 of 109 transformants cured, Figure 1B *, top row*) while expression of wild-type Apj1 restored Hsp104 curing (111 of 112 transformants cured). Likewise, as previously reported, Apj1-161 was nearly as good as full-length protein with 43 of 61 transformants cured (Berger et al., 2020).

We next tested a series of C-terminal truncations and internal deletions to determine what regions of Apj1-161 might be important for Hsp104 curing. We deleted the QA repeat region in the context of Apj1-161 (Apj1-161_ΔQA_) and found that loss of this region dramatically impaired curing (14 of 70 transformants cured). In contrast, a construct lacking the SNF region (Apj1-110) retained its curing activity (39 of 44 cured), while the same construct without the QA region lost curing ability (Apj1-110_ΔQA_; 3 of 40 cured). Further C-terminal truncations Apj1-101 and Apj1-90, the latter lacking the entire GF region, retained curing activity with 39 of 48 and 64 of 71 transformants cured, respectively. Apj1-90 was the minimal construct that supported curing as further truncations and deletions resulted in impaired curing but were unstable.

To test whether Apj1 cooperates with Hsp70 in this process, we expressed Apj1-161 with the point mutation H_34_→Q that disrupts J-domain function in other JDPs (Tsai and Douglas, 1996). As expected, Apj1-161_H34Q_ failed to support curing (Figure 1B, 0 of 60 transformants cured), indicating that J-domain activity is critically required and, logically, that Apj1 is acting in concert with an Hsp70 chaperone in curing. Since the J-domain must be functional, we next asked whether there is anything particular about Apj1’s own J-domain that may be important in this process. We replaced Apj1’s J-domain with that of the more distantly related Class C JDP Jjj1 to create a chimeric protein (JA-161) possessing residues 1–68 of Jjj1 (the J-domain) followed by residues 71–161 of Apj1. Surprisingly, JA-161 was unable to support efficient prion curing (3 of 48 transformants cured), despite being highly functional in other assays, as described in the next section.

In summary, these results demonstrate that efficient Hsp104 curing of strong [*PSI* ^+^]^Sc4^ can be accomplished by a minimal construct consisting only of Apj1’s J-domain and the immediate C-terminal region encompassing residues 72–90 that includes the QA repeat region, whereas the GF and SNF regions are completely dispensable. Additionally, the J-domain must be functional but cannot be simply replaced by any cytosolic J-domain.

### 3.3 A functional J-domain with Apj1’s GF allows the growth of *sis1*-Δ cells

**FIGURE 2:**
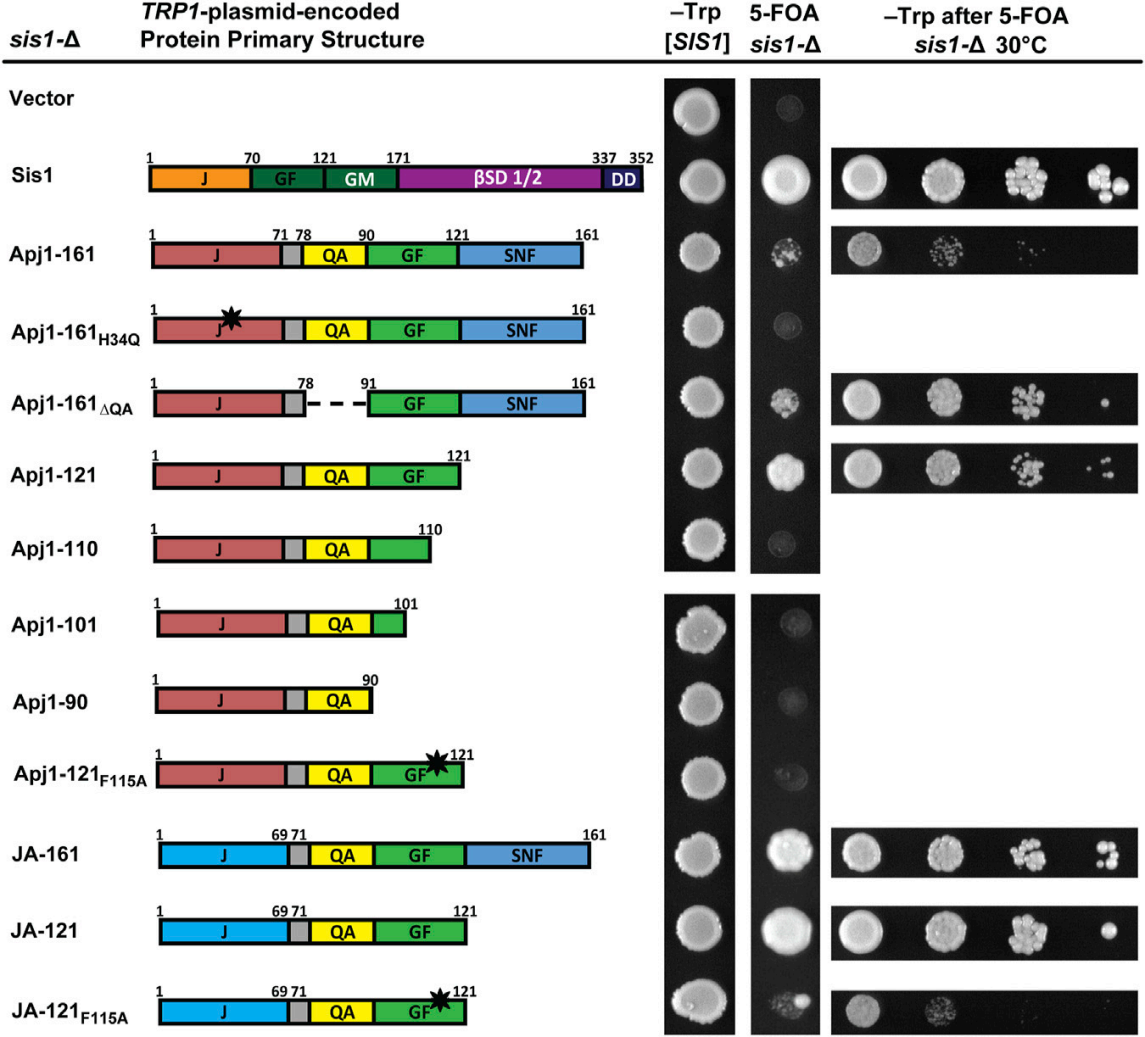
Constructs of Apj1 are capable of replacing Sis1 to maintain cell viability. Comparison of primary protein structures of wild type Sis1 and Apj1 constructs used in this assay. Protein region abbreviations are described in Figure 1. Dashed lines indicate where a region has been deleted. The black star indicates a point mutation. *sis1-∆* cells expressing Sis1 from a *URA3*-marked plasmid were transformed with a second *TRP1*-marked plasmid carrying either Sis1 or various Apj1 constructs and spotted onto solid synthetic medium lacking tryptophan (–Trp) or containing 5-FOA and incubated for 2 days or 4 days respectively at 30°C (*middle columns*). Note: a single large colony in the JA-121_F115A_ sample is anomalous, and cells from this colony were not used for additional tests. For constructs able to rescue growth of *sis1-*Δ cells on 5-FOA, plasmid-shuffled cells were counted and suspended to equivalent densities. Cells were then serially diluted 10-fold, spotted in 5 μL drops on medium selective for the *TRP1* marker (*right column*), and incubated for 3 days at 30°C. Representative examples are shown from the same experiment, with all experiments being repeated a minimum of three times.

Given that Apj1 and Sis1 are both involved in Hsp104 curing, that overexpression of either protein could complement a deletion of the other in this process (Astor et al., 2018), and that Sis1-121 is sufficient to both maintain cell viability and propagate strong variants of both the prions [*PSI* ^+^] and [*RNQ* ^+^] (Harris et al., 2014), we were surprised to find that the GF region of Apj1 was unimportant for curing. We wondered if constructs of Apj1 containing the GF region, which has some sequence elements similar to Sis1, could compensate for Sis1 in this or other functions. To test this hypothesis, we asked whether truncated or mutated constructs of Apj1 could replace Sis1. Surprisingly, Apj1-161 partially rescued *sis1-∆* cells, though with a significant slow-growth phenotype (Figure 2). As expected, rescue was J-domain-dependent as Apj1-161_H34Q_ did not support growth. In contrast to its effect on Hsp104 curing, deletion of the QA region did not impair the ability of Apj1-161 to rescue *sis1-∆*. However, the GF region was essential as all C-terminal truncations that lack all or part of Apj1’s GF region failed to complement *sis1*-Δ (Figure 2).

A recent analysis of the GF region of Sis1 identified residues that were critical for cell viability within the sequence DAFNIFSQFF (Ciesielski, et al., 2024). The F two positions preceding the QFF was identified as a key residue; when Sis1-121 is expressed as the sole version of Sis1, mutation of this phenylalanine to alanine was lethal. To see if Apj1’s GF region may function like Sis1’s, we tested whether the loss of the analogous phenylalanine in the DLFAQFF would affect the ability of Apj1-121 to rescue Sis1; it did lose all ability to do so (Figure 2).

To assess whether Apj1’s J-domain was specifically important for the rescue of Sis1, as it was for Hsp104 curing, we tested the JA-161 construct containing Jjj1’s J-domain in place of Apj1’s. Surprisingly, JA-161 rescued *sis1-∆* better than Apj1-161. Given this result, we surmised that a shorter construct, ending on residue 121, might be superior. Indeed, JA-121 rescued *sis1*-Δ better than all other constructs and nearly to the same level as wild-type Sis1. As expected, the F_115_→A mutation rendered the JA-121 construct almost completely nonfunctional, again underscoring the importance of this residue (Figure 2). The rescue of *sis1*-Δ by various constructs at 34°C and 37°C is less robust but revealed greater differences among constructs with the same patterns (Supplemenatry Figure S1). Combined, these results indicate that Apj1’s GF region is surprisingly similar in function to that of Sis1, despite its presence in a Class A JDP that is evolutionarily derived from Ydj1, the canonical Class A JDP in yeast.

To determine if the lack of functionality of some Apj1 constructs might be due to low protein expression, we tagged various constructs with a 3XFLAG tag. Tagged constructs behaved similarly to their untagged counterparts in functional assays excluding Apj1-161, where the tag disrupted the ability to support Hsp104 curing as effectively. All tagged constructs were expressed at similar or higher levels than Apj1-161 (Supplemenatry Figure S2). These results indicate that the lack of functionality of Apj1-161_H34Q_, Apj1-161_ΔQA_, Apj1-110_ΔQA_, Apj1-121_F115A_, and JA-121_F115A_ is not likely due to low protein expression or poor protein stability.

### 3.4 Apj1 constructs can propagate multiple prions in place of Sis1

**FIGURE 3:**
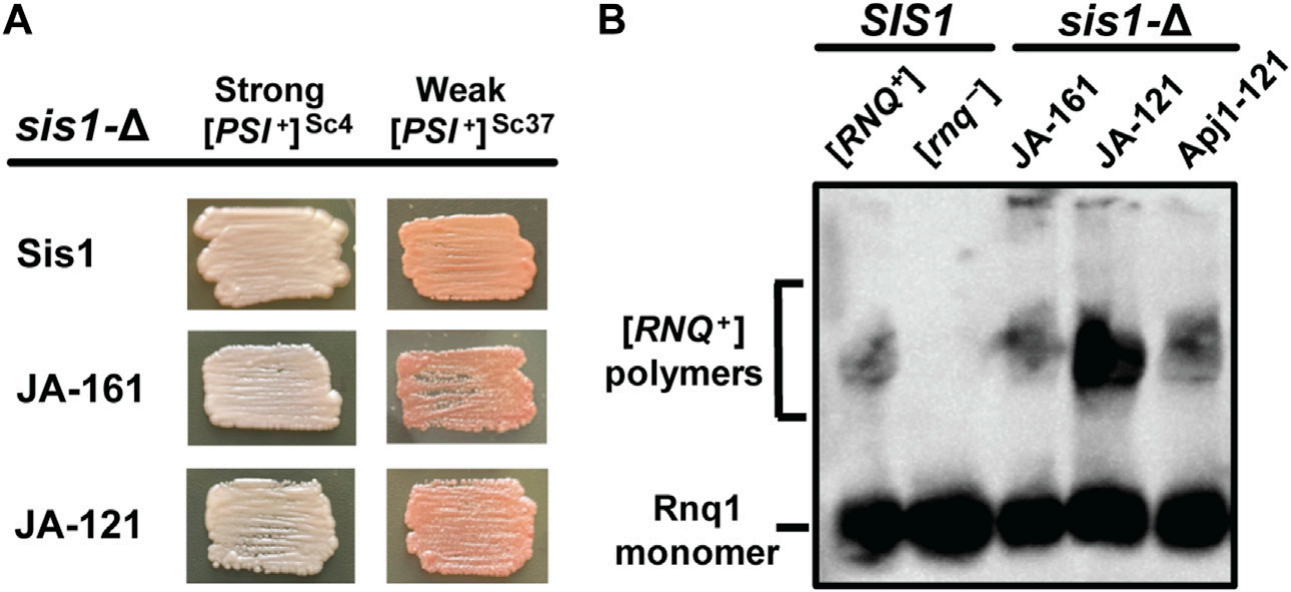
Apj1 constructs containing its GF region can replace Sis1 in prion propagation. *sis1-*Δ cells expressing Sis1 from a *URA3*-marked plasmid and bearing [*PSI* ^+^]^Sc4^, [*PSI* ^+^]^Sc37^, or [*RNQ* ^+^]^STR^ were transformed with plasmids expressing Sis1 or Apj1 constructs. Transformants were plated onto 5-FOA medium to counter-select against the *URA3*-marked Sis1 plasmid and repatched two times on selective medium to allow time for prion loss. **(A)** To evaluate the continued presence of [*PSI* ^+^], color phenotype assays (*n* ≥ 10 for each construct) are shown for representative colonies. All transformants maintained [*PSI* ^+^] in every case. **(B)** [*RNQ* ^+^] maintenance and loss were evaluated by SDD-AGE, followed by immunoblotting using a Rnq1-specific antibody for *n* = 8 transformants per construct with all transformants maintaining the prion. Detergent-resistant aggregates are resolved from monomeric Rnq1 using this method. A representative blot showing one sample for each construct in the *sis1*-Δ background is shown with samples from control (*SIS1*) [*RNQ* ^+^] and [*rnq* ^−^] cells included for comparison.

Because Apj1 constructs bearing Apj1’s GF region can rescue the cell viability of a *sis1*-Δ strain, we next asked if these constructs could replace Sis1 in prion propagation. We examined the ability of various constructs that rescue *sis1*-Δ to replace Sis1 in the propagation of three distinct, well-studied prions: a strong variant of [*PSI* ^+^] ([*PSI* ^+^]^Sc4^), a weak variant of [*PSI* ^+^] ([*PSI* ^+^]^Sc37^), and a strong variant of [*RNQ* ^+^] ([*RNQ* ^+^]^STR^) (Harris et al., 2014). Both JA-121 and JA-161 were capable of maintaining all three prions (Figure 3). Apj1-121, which was less able to compensate for Sis1 in viability, was unable to do so in [*PSI* ^+^] strains, almost certainly due to the well-documented toxicity of [*PSI* ^+^] in strains with poor Sis1 function (Kirkland et al., 2011; Harris et al., 2014). We then asked if Apj1-121 could support the propagation of [*RNQ* ^+^]^STR^ as its presence is not known to be toxic to cells and thus does not require robust Sis1 activity to support cell growth. Apj1-121 was able to rescue viability in place of Sis1 in a [*RNQ* ^+^]^STR^ strain; in that strain, it supports [*RNQ* ^+^]^STR^ propagation (Figure 3B). This result is significant because [*RNQ* ^+^] is well-known to be extremely sensitive among prions to alterations in the GF region of Sis1 (Stein and True, 2014; Killian et al., 2019), and so the ability of Apj1-121 to propagate this prion again underscores the functional overlap of Apj1’s GF region with that of Sis1.

## 4 Discussion

Our analysis of the region between the J-domain and the client-binding domain in the Class A JDP Apj1 led to unexpected findings. First, the QA segment that is sufficient (in conjunction with the J-domain) for promoting Hsp104-dependent prion curing differs in sequence from sequences previously implicated in this process. Second, although much less glycine rich than is typical, the middle “GF” segment of this region has a striking sequence and functional similarity to “Helix V” of other Class A/B JDPs. Together, these results point to the divergence in sequence and function of this region, while maintaining core activity.

### 4.1 Apj1’s QA region and prion loss

**FIGURE 4:**
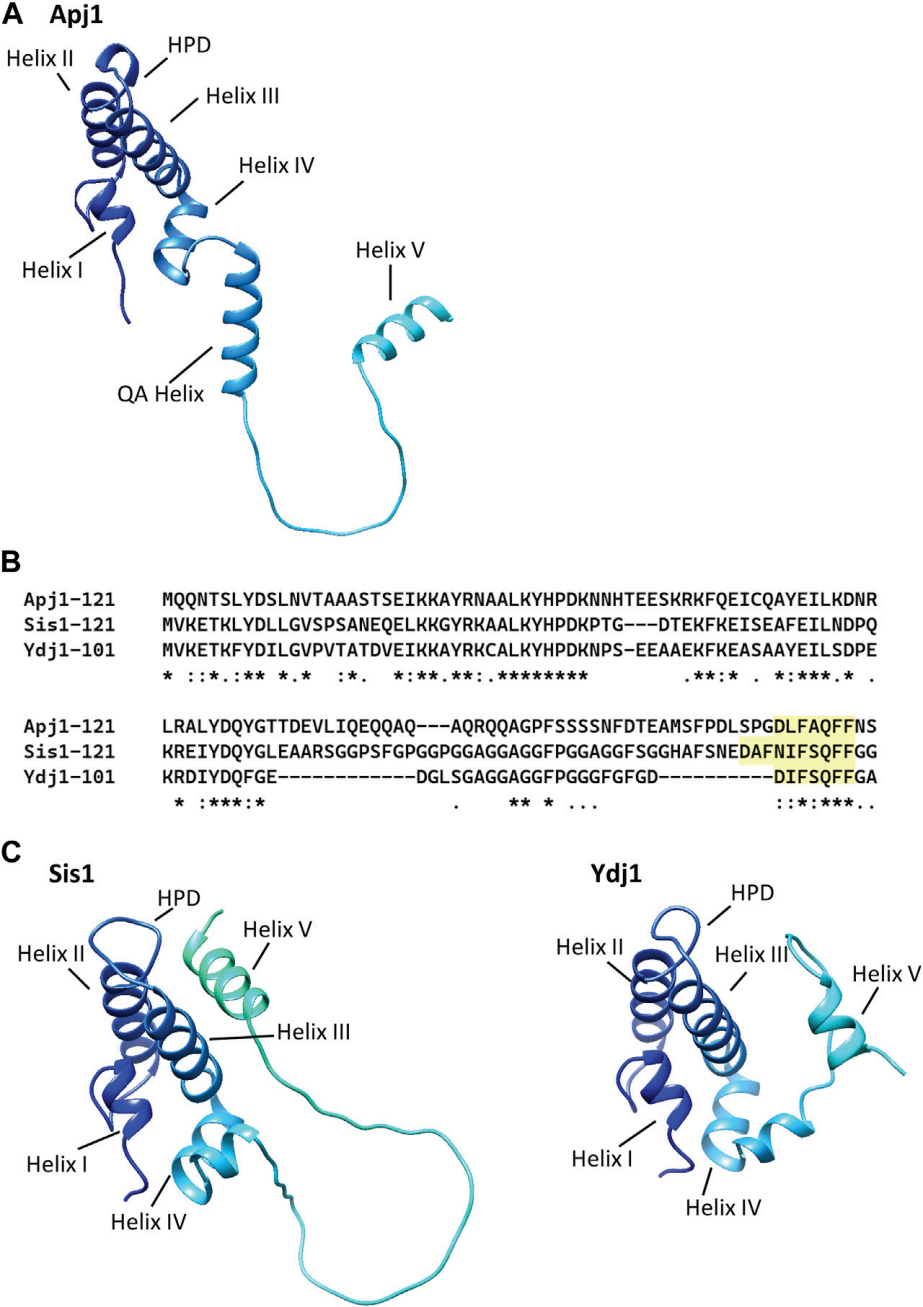
AlphaFold models of Apj1, Sis1, and Ydj1 show a conserved Helix V in multiple positions and an elongated “QA” helix in Apj1. AlphaFold models of the J-domain and adjacent sequences of Apj1, Sis1, and Ydj1 from strain S288c. The four-helical J-domain is shown in dark blue in the upper left of each structure. **(A)** AlphaFold prediction for Apj1-121. Residues 75–89 form an extended helix, called the “QA Helix”, that extends away from the J-domain. The GF region forms an extended loop terminating in Helix V (residues 111-119) which is away from the J-domain. **(B)** Multiple sequence alignment (Clustal Omega) of the J-domain and adjacent Gly-rich regions of Apj1, Ydj1, and Sis1 (Madeira et al., 2022). The conserved sequences with homology to Ydj1’s DIFSQFF region are highlighted. **(C)** AlphaFold predictions for Sis1-121 (left) and Ydj1-101 (right). In Sis1-121, Helix V interacts with Helices II and III and sterically blocks the HPD motif; this location for Helix V is congruent with NMR data for Sis1 (Ciesielski, et al., 2024) and similar to that observed in NMR structures of the human Sis1 ortholog DNAJB1 (Faust et al., 2020). In Ydj1-101, Helix V is in the foreground on the right side, away from Helix II and III and the HPD motif.

The regions between the end of Helix IV of the J-domain and the end of the QA region are sufficient for Apj1 to function in Hsp104-dependent prion loss. The Apj1 J-domain must be functional and cannot simply be replaced by any J-domain that functions with the same Hsp70 (*i.e.*, Jjj1, which also functions with Ssa Hsp70s). However, such Jjj1-J-domain-Apj1 chimeric constructs are functional in other assays, substituting for Sis1 for cell growth and maintaining prions. AlphaFold predictions (Jumper et al., 2021; Varadi et al., 2022) show the QA region as part of a helix (Figure 4A). Although a very short helical region is thought to extend beyond Helix IV of the J-domain in many Class A/B JDPs, a helix of this length (15 residues) is unusual. While the GF region of Apj1 was dispensable for curing, we previously showed that GF region of Sis1 is essential for Hsp104 prion elimination (Astor et al., 2018). Therefore, Apj1 and Sis1 act through different parts of their respective proteins in supporting prion curing. Since there is little, if any, sequence similarity between the required regions, they likely do so by different, although still undefined, mechanisms.

### 4.2 Apj1’s GF region in relation to Sis1 function and prion propagation

Finding that the presence of the GF region of Apj1 correlates with the ability of short Apj1 fragments to allow the growth of cells lacking Sis1 is, at first glance, surprising and raises many questions regarding GF region functionality, as discussed below. However, importantly, related to prion biology, this ability allowed testing of prion maintenance. The robustness of prion maintenance by Apj1 fragments was striking. While maintenance of strong [*PSI* ^+^] is not unusual if viability is maintained (Killian and Hines, 2018), weak [*PSI* ^+^] maintenance is extraordinary as the weak [*PSI* ^+^] variant [*PSI* ^+^]^Sc37^ used here cannot be maintained by Sis1-121 in the same yeast genetic background (Harris et al., 2014). Likewise, the ability of Apj1-121 and JA-121 to maintain [*RNQ* ^+^] is equally remarkable because the [*RNQ* ^+^] requirement for Sis1’s GF region is extreme: of at least six distinct variants of [*RNQ* ^+^] that have been well-characterized, no variant has yet been identified that can propagate without the expression of Sis1’s GF region in cis with a functional J-domain (Lopez et al., 2003; Stein and True, 2014; Killian et al., 2019). The ability of JA-121 to propagate all three prions indicates that this heterologous combination of a J-domain and GF region, neither of which come from Sis1, is more functional in prion propagation than the combination of the same domains from Sis1 (Sis1-121).

We did not expect that short Apj1 fragments would support the growth of *sis1*-Δ cells as it was not initially apparent that there was significant sequence similarity between the regions of Sis1-121 and Apj1. In addition, full-length Apj1 overexpression does not rescue *sis1*-Δ (Sahi and Craig, 2007). However, recent experiments with Ydj1 are informative relative to our observations regarding Apj1 reported here. Although Ydj1 cannot normally substitute for Sis1, a single residue substitution at the junction of the J-domain and the adjacent GF region (G_70_→N) results in a gain in function, allowing the growth of *sis1*-∆ cells (Schilke et al., 2017). The Ydj1 J-domain-GF fragment bearing the same mutation but lacking the C-terminal domains (Ydj1-109_G70N_) actually supports better growth, suggesting that perhaps these domains sterically interfere (Schilke et al., 2017; Ciesielski, et al., 2024). Such interference may also explain the lack of rescue by full-length Apj1. Interestingly, a construct having a deletion of the QA region and a construct with the J-domain swapped both better support growth, consistent with a functional influence of the structure of the junction between the J-domain and its adjacent segment.

The similarity between the Apj1 GF sequence (DLFAQFF) and the important Ydj1 sequence (DIFSQFF) is more striking than that with the Sis1 region (Figure 4B), particularly regarding the spacing of negatively charged and hydrophobic residues (Ciesielski, et al., 2024). In all three, formation of a helix is predicted—generally referred to as Helix V (Figure 4C)—and confirmed by biophysics analysis for Sis1 and Ydj1 (Ciesielski, et al., 2024). However, what Helix V is doing in the context of the short N-terminal segments of Class A/B JDPs is not well understood. Experimental evidence has led to the hypothesis that Helix V plays a role in modulating the substrate binding cycle of Hsp70 in both Class A and B JDPs (Ciesielski, et al., 2024) while also playing an important intramolecular regulatory role in eukaryotic Class B JDPs (Yu et al., 2015a; Yu et al., 2015b; Faust et al., 2020; Abayev-Avraham et al., 2023).

### 4.3 The function of Apj1’s SNF region is unknown

We found no critical role for the SNF region in any of our assays. We had noted at the outset of our investigation its similarity to known yeast prion-forming domains (PrDs) which could potentially allow for such a region to bind favorably to the ends of amyloid fibers, resulting in prion curing by a capping mechanism that disrupts native prion elongation. Fully half of the 22 proteins from the “Alberti set” of *S. cerevisiae* amyloid-forming proteins have PrDs enriched in S, N, and G—some are rich in F and all are low in charged residues and proline (Alberti et al., 2009). Indeed, the amyloid prediction programs *RFAmyloid* and *AmylPred2* predict that this region has the propensity to form amyloid. Notably, this composition also closely resembles the G-, F- and S-rich region of DNAJB6 in which numerous serines were found to directly bind Aβ (1–40) oligomers and suppress fibril formation (Österlund et al., 2020). However, its presence in the Apj1 sequence and possible role in any amyloid-related biology remain a mystery.

### 4.4 Concluding remarks and remaining questions

Our investigation has uncovered several unexpected functional capabilities for Apj1’s J-domain and proximal regions, revealing a far more significant intertwinement of the structures and functions of JDPs than has been previously appreciated. These results are also significant in advancing our understanding of prion-chaperone interactions as they bring us closer to understanding the specific amino acid sequences required for conformer-specific prion propagation and elimination in yeast, and thereby help inform JDP-amyloid interactions in humans.

Nevertheless, unanswered questions remain. The mechanism by which Hsp104 eliminates prions upon overexpression is still the subject of considerable debate despite years of research (Helsen and Glover, 2012; Winkler et al., 2012; Park et al., 2014; Ness et al., 2017; Zhao et al., 2017; Cox and Tuite, 2018; Matveenko et al., 2018). In these debates, the critical roles played by JDPs—both Sis1 and Apj1—remain to be considered. We have here established that Apj1 collaborates with Hsp70 in this process, but what is the role of the QA region/helix in prion curing? What residues within this region are important? Likewise, we have found that Apj1’s GF region, when paired with a functional J-domain, is competent to replace Sis1 to maintain cell viability and prion propagation. What is the biochemical mechanism that is critical for this process but cannot be done by other JDPs present in the cytosol? Again, it is clear that Hsp70 is involved, but what is accomplished by the J-domain-GF fragments of these proteins beyond Hsp70 ATPase stimulation?

## Data Availability

The raw data supporting the conclusion of this article will be made available by the authors, without undue reservation.
