# Supplementary material for: Unique characteristics of the J-domain proximal regions of Hsp70 cochaperone Apj1 in prion propagation/elimination and its overlap with Sis1 function
Source: Front Mol Biosci. 2024 Apr 24;11:1392608. doi: 10.3389/fmolb.2024.1392608 (PMC11078019; doi:10.3389/fmolb.2024.1392608)
Supplement: Supplementary file 1 [file DataSheet1.docx]

**SUPPLEMENTAL MATERIAL**

**Table S1. Plasmids used in this study.**

|  |  | | |  |  |
| --- | --- | --- | --- | --- | --- |
|  |  |  |  |  |  |
| Plasmid | Promoter | Marker | Copy number | Source |  |
| pRS313*-SIS1 SIS1* | *SIS1* | *HIS3* | CEN, low | (Yan and Craig, 1999) |  |
| pRS314*-SIS1 SIS1* | *SIS1* | *TRP1* | CEN, low | (Yan and Craig, 1999) |  |
| pRS424*-GPD APJ1* | *GPD* | *TRP1* | 2μ, high | (Sahi and Craig, 2007) |  |
| pRS424*-GPD APJ1-161* | *GPD* | *TRP1* | 2μ, high | (Sahi and Craig, 2007) |  |
| pRS424*-GPD APJ1-161_H34Q_* | *GPD* | *TRP1* | 2μ, high | This study |  |
| pRS424*-GPD APJ1-161*_ΔQA_ | *GPD* | *TRP1* | 2μ, high | This study |  |
| pRS424*-GPD APJ1-121* | *GPD* | *TRP1* | 2μ, high | This study |  |
| pRS424*-GPD APJ1-121_F115A_* | *GPD* | *TRP1* | 2μ, high | This study |  |
| pRS424*-GPD APJ1-110* | *GPD* | *TRP1* | 2μ, high | This study |  |
| pRS424*-GPD APJ1-110*_ΔQA_ | *GPD* | *TRP1* | 2μ, high | This study |  |
| pRS424*-GPD APJ1-101* | *GPD* | *TRP1* | 2μ, high | This study |  |
| pRS424*-GPD APJ1-90* | *GPD* | *TRP1* | 2μ, high | This study |  |
| pRS424*-GPD JA-161* | *GPD* | *TRP1* | 2μ, high | This study |  |
| pRS424*-GPD JA-121* | *GPD* | *TRP1* | 2μ, high | This study |  |
| pRS424*-GPD JA-121_F115A_* | *GPD* | *TRP1* | 2μ, high | This study |  |
| pRS424*-GPD APJ-3XFLAG* | *GPD* | *TRP1* | 2μ, high | This study |  |
| pRS424*-GPD APJ1-161-3XFLAG* | *GPD* | *TRP1* | 2μ, high | This study |  |
| pRS424*-GPD APJ1-161_H34Q_ -3XFLAG* | *GPD* | *TRP1* | 2μ, high | This study |  |
| pRS424*-GPD APJ1-161*_ΔQA_*-3XFLAG* | *GPD* | *TRP1* | 2μ, high | This study |  |
| pRS424*-GPD APJ1-110-3XFLAG* | *GPD* | *TRP1* | 2μ, high | This study |  |
| pRS424*-GPD APJ1-101-3XFLAG* | *GPD* | *TRP1* | 2μ, high | This study |  |
| pRS424*-GPD APJ1-90-3XFLAG* | *GPD* | *TRP1* | 2μ, high | This study |  |
| pRS424*-GPD APJ1-121_F115A_ -3XFLAG* | *GPD* | *TRP1* | 2μ, high | This study |  |
| pRS424*-GPD APJ1-110*_ΔQA_*-3XFLAG* | *GPD* | *TRP1* | 2μ, high | This study |  |
| pRS424*-GPD JA-161-3XFLAG* | *GPD* | *TRP1* | 2μ, high | This study |  |
| pRS424*-GPD JA-121-3XFLAG* | *GPD* | *TRP1* | 2μ, high | This study |  |
| pRS424*-GPD JA-121_F115A_-3XFLAG* | *GPD* | *TRP1* | 2μ, high | This study |  |
| pRS416*-GPD-HSP104* | *GPD* | *URA3* | CEN, low | (Sporn and Hines, 2015) |  |

**Figure S1**

**
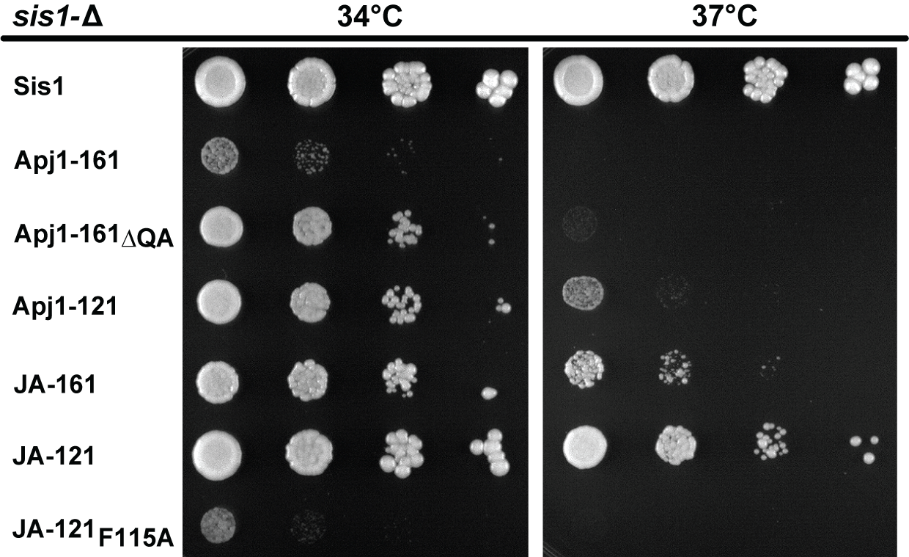
**

**Supplemental Figure 1. Apj1 constructs differentially allow growth of *sis1*-Δ cells at elevated temperatures.** *sis1-∆* cells expressing Sis1 from a *URA3*-marked plasmid were transformed with a second *TRP1*-marked plasmid carrying expressing either Sis1 or various Apj1 constructs and incubated for 3 days at 30°C on medium containing 5-FOA that counter-selects against the *URA3*-marked plasmid. After plasmid shuffling, cells were suspended to equivalent densities. Cells were then serially diluted 10-fold and spotted in 5 μL drops on selective medium. Representative examples are shown for cells grown at 34°C (left) or 37°C (right) from the same experiment with all experiments repeated a minimum of three times.

**Figure S2**

**
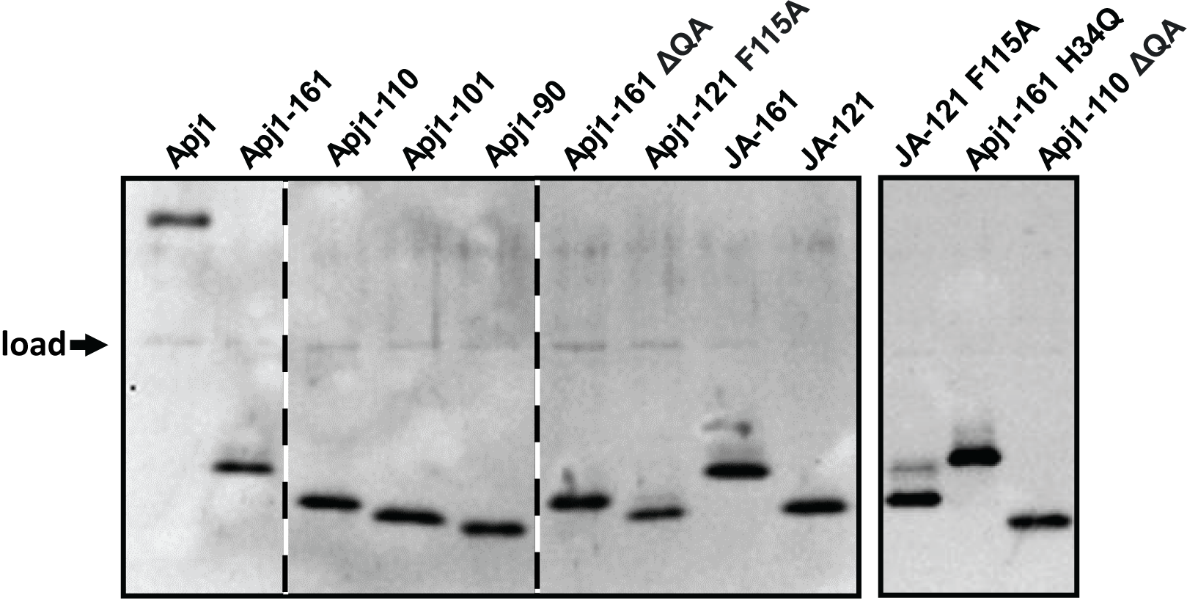
**

**Supplemental Figure 2. Expression levels of tagged constructs.** Lysates of cells bearing 3XFLAG-tagged constructs were resolved by SDS-PAGE and subjected to immunoblot analysis using antibody raised against the 3XFLAG tag. Load control shown is a nonspecific protein cross-reacting with antibody. Dashed line separates lanes taken from different parts of the same gel. Solid black outlines denote separate gels.
