# Supplementary figures and images for: Unique characteristics of the J-domain proximal regions of Hsp70 cochaperone Apj1 in prion propagation/elimination and its overlap with Sis1 function
Source: Front Mol Biosci. 2024 Apr 24;11:1392608. doi: 10.3389/fmolb.2024.1392608 (PMC11078019; doi:10.3389/fmolb.2024.1392608)

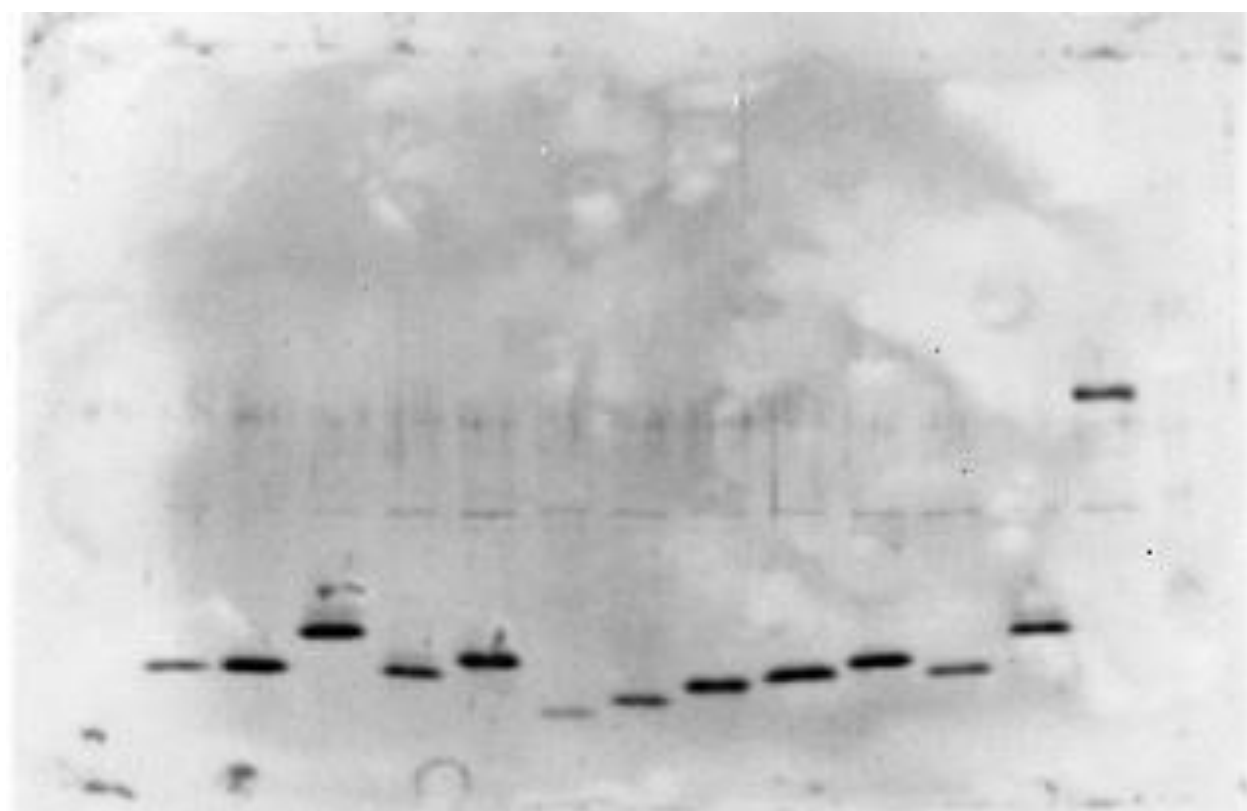

Supplement: Supplementary file 2 [file Image1.pdf]
